# Supplementary material for: The Open Cell Form of 3D-Printed Titanium Improves Osteconductive Properties and Adhesion Behavior of Dental Pulp Stem Cells
Source: Materials (Basel). 2021 Sep 15;14(18):5308. doi: 10.3390/ma14185308 (PMC8467079; doi:10.3390/ma14185308)
Supplement: Supplementary file 1 [file materials-14-05308-s001.zip › materials-1334974-supplementary.pdf]

materials

Article

The Open Cell Form of 3D-Printed Titanium Improves Osteconductive Properties and Adhesion Behavior of Dental Pulp Stem Cells

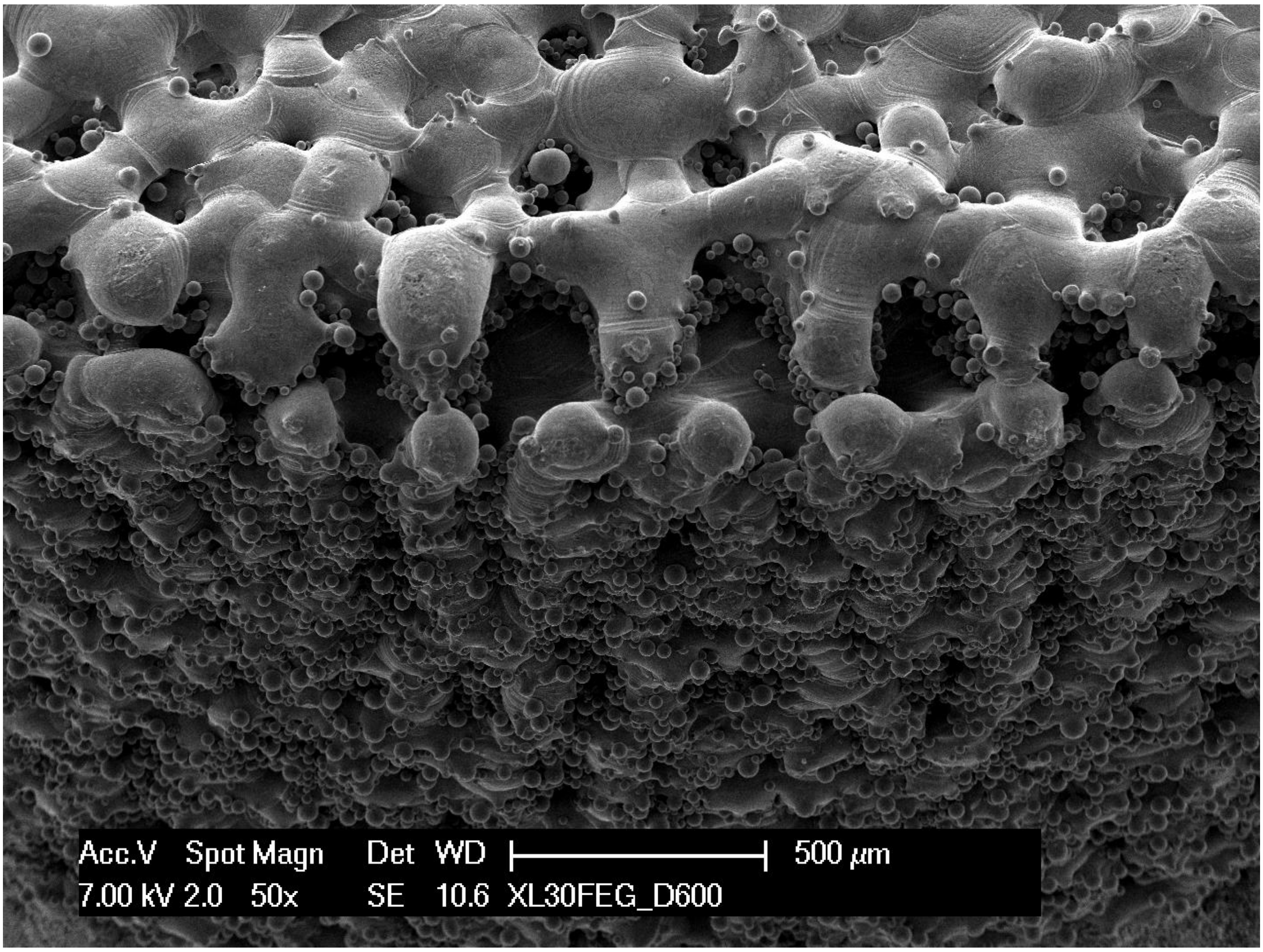

Figure S1. SEM image of Titanium A showing the cross-sectional view of the sample evidencing the interconnected pores. Magnification: 50×.
